# Trust in the Third Space of Science Education

**DOI:** 10.1007/s11191-022-00356-w

**Published:** 2022-07-19

**Authors:** Maurizio Toscano, Victoria Millar

**Affiliations:** Melbourne Graduate School of Education, University of Melbourne, Melbourne, Victoria 3010 Australia

## Abstract

This paper investigates rationality and its relationship to trust in science in the context of three proposed spaces of science education: the formal, informal and casual. It begins with the place of science as a trusted institution and its role in formal and informal education across the world. Through educational systems, we have come to trust that students are being educated about science and its trustworthiness. However, formal and informal education spaces are not the only spaces in which individuals and society seek science understanding. While the science education literature has long concerned itself with science education in these spaces, this paper proposes a third space, the casual space. The casual space is decentralised and provides access to a range of norms and explanations about the world. We investigate how each of the formal, informal and casual spaces privileges particular forms of rationality as a means for understanding trust in science in each of these spaces. This paper considers the implications for education’s response to the challenge of equipping students to make rational judgements about science.

## Introduction

Science has remained an essential component of a modern, technological and global society. It contributes to our understanding of the world and has succeeded in improving human wellbeing, particularly through medical and technological advances. Science also continues to contribute to a culture that has come to value expansive and liberating scientific insights and perspectives. Consequently, science has earned the status of a trusted institution within society (Hmielowski et al., 2014a, 2014b). Trust in science has emerged from its reliability in providing us with objective, rational and applicable accounts of the world that are also amenable to renewal through the methods of science (Grasswick, 2010).

Yet the success of science has also been accompanied by an increase in the volume, complexity and specialisation of scientific knowledge, with the consequence that a comprehensive view of science eludes the specialist and layperson alike (Hendriks et al., 2016). There are consequences insofar as scientific knowledge has become less accessible and comprehensible to the general public. As the public is faced with increasingly complex ideas, models and explanations, a greater level of trust in experts, the institutions responsible for scientific progress and those that communicate science is required. The question of who to trust depends on having the critical and analytic skills required to evaluate complex rational science. This is made increasingly difficult as science and trust in science are increasingly challenged. This is evident in the long-standing debates in the public about climate change and more recently in relation to the COVID-19 pandemic. While much of the public still appreciates science (Castell et al., 2014; European Commission, 2013) and believe that scientists have good intentions (Besley, 2014; European Commission, 2013), there is evidence to suggest that trust in public institutions, including science, is waning (Borgonovi, 2012).

Trust in science may depend upon more than the content and the means by which the truthfulness and reliability of science are established and justified. Hence, scientific forms of rationality—that is, the means of establishing objective and justified true beliefs—may at times, and in different contexts, be insufficient or unnecessary to establish trust. For instance, the general public may trust science and accept the conclusions and claims of science (e.g. we must reduce emissions of carbon dioxide to mitigate the deleterious effects of climate change) without any knowledge or understanding on their part for how such claims are established and justified. On other occasions, people may accept the scientific claims (e.g. vaccines work), but may be less trusting of science acting as an institution that is not immune from bias and political and economic interests (e.g. the influence of pharmaceutical companies on regulatory bodies). This suggests that trust is highly contextual and subject to the kinds of judgements people make on any given occasion and within a particular context.

We may legitimately question the degree to which, and the means by which, the rationality of science can maintain the kind of trust it has enjoyed to date—especially in times when the public must contend with complex science-informed issues (e.g. climate change) or events characterised by rapidly evolving scientific understanding (e.g. COVID-19 pandemic). This entails examining the contexts or spaces in which science and scientific knowledge is communicated, taught and applied. It also involves examining the kinds of practices and ends for which the rationality afforded by science is useful, reliable and therefore worthy of trust. Yet we must also consider forms of rationality other than those supported by science that nevertheless promise and deliver reliability, and therefore have the potential to generate forms of trust complementary to (or in competition with) trust in science.

Maintaining trust in science relies on science meeting societal expectations while adhering to science’s characteristic norms and values. Such societal expectations are not free-floating. They are shaped by the spaces in which interactions with science arise in a structured way (say through formal science education) or in an emergent way (e.g. through mass media in a time of crisis like a global pandemic). Some of these spaces provide access to reliable and trustworthy science, for example, the science classroom, whereas others, like social media platforms, provide access to a plethora of diverse norms and forms of rationality. How then does someone go about making judgements of the trustworthiness of scientific information, and whether on a given occasion science is succeeding in meeting the expectations set out by society?

In this paper, we examine the spaces in which science is socially significant with respect to establishing trust in science. These include spaces like schools and museums, but also the private space of the home or the Internet. We examine these in relation to the part that science education may play in foregrounding different forms of rationality, including those that are more characteristic of science. This will enable a better understanding of the roles that formal and informal science education have in providing students with the ability to make judgements about the trustworthiness of science. It also permits us to explore the limitations of formal and informal science education and communication, as well as the potential or otherwise of developing trust in science in diverse contexts.

We do so by proposing a model of three spaces in which the general public engage with and are educated in scientific knowledge and practices in ways that may promote trust in science. The first two are the familiar spaces of formal and informal science education, while the third —casual space—we argue is necessary to account for a different array of forms of rationality. This casual space affords alternative arrangements of societal expectations that may affect public trust in science in ways that are less prominent within the institutional, centralised and regulated spaces of formal and informal science education.

This casual space shows up in the public sphere of everyday social interactions in which scientific knowledge, values, norms and utility sit alongside a plurality of other forms of rationality and systems and practices of meaning making. It includes life within a local and global community, life within a family or social group, and life in a complex of material, embodied and virtual experiences. Indeed, increasingly, this space is experienced online. In 2012, a US survey found that 42% of respondents mentioned that the Internet was their primary source of information about science and technology (Besley, 2014). For specific science issues, the survey found that 63% respondents made use of the Internet (Besley, 2014). In the UK, a similar survey found 67% of young people used the Internet to look for information about science (Wellcome Trust, 2013). Similar results have been found for Europe (European Commission, 2013) and Australia (Department of Business and Innovation, 2012).

The casual space is one that has not had a great deal of attention in science education despite this space being increasingly used as a source of information and discourse about science. Hence, this paper seeks to distinguish it from the formal and informal spaces in order to determine whether it affords different opportunities for trust in science relative to the other spaces, and its call to reconceptualise the role of science education outside institutions.

In this paper, we ask: What forms of rationality are taken up in the three spaces of science education and what kind and degree of trust in science do they support? We do this to understand how judgements about the trustworthiness of science are situated amongst other assessments of personal and collective meaning. In response to these questions, the following sections explore the concepts of trust and trust in science, and what makes something trustworthy. Following this, we introduce a framework for differentiating forms of rationality before describing and justifying a proposed model of formal, informal and casual science education. Once this grounding has been explained, we explore the forms of rationality at play in each of the three spaces.

## What is trust?

Trust is a belief that other agents will act in a predictable way and fulfil their obligations without special sanctions (Coleman, 1988). It is an attitude directed towards people in general as well as groups of people or institutions. Trust is relational, and therefore has a role to play in establishing and maintaining social cohesion and stability. Society, institutions, families and other collectives—insofar as they depend on enduring and stable identities—require trust to function as a way of holding their constituents together via mutual agreements that set promises with an expectation that they are met. Furthermore, such networks of committed relationships with “shared norms, values and understandings that facilitate co-operation within or among groups” (Scrivens & Smith, 2013, p. 18) acquired through social networks and public engagement (Putnam, 2000) also constitute forms of social capital upon which individuals and communities can rely. Social capital is beneficial to prosperity and democratic stability (Fuller, 2014; Niedlich et al., 2021; Putnam, 2000), yet it relies upon environments in which a store of “social capital” (norms, values, practices) can be called upon reliably to set and to serve people’s goals and interests.

Within the complexity of society, trust is important for individuals as well as institutions, and is a means for responding to complexity and mitigating risk as people are faced with making choices about many things that fall outside their expertise (Blöbaum, 2016). Trust therefore provides a means for managing risk associated with decisions (Giddens, 2013). So, as Blöbaum (2016, p. 5) puts it: “in the act of trust, a particular future is anticipated, and access is opened up to information and events outside the realm of one’s experience”. Where an institution is offering knowledge, the trust in that knowledge generates what may be called epistemic trust (Resnik, 2011; Sperber et al., 2010). Epistemic trust in science requires trust in the educative spaces in which science is accessed and learnt.

What makes an individual or an institution trustworthy; especially considering evidence to show that trust, alongside social capital and cohesion, is declining (Oreskes, 2019; Putnam, 2000; Twenge et al., 2014)? Trust is achieved by doing “the right thing at the right time for the right reasons, and the action should have its intended effect” (D’Olimpio, 2018). Generally, we have trust in those who, and in that which, promises and/or provides us with an account of the world and an orientation towards it that is reliable and supports our purposeful and effective judgements and actions. What this means in practice is that we have in what/who is trusted a source of insight (knowledge, understanding, wisdom, experience, etc.) for decision-making and directed action in a world that is otherwise too complex or too risky to navigate alone. Trust therefore speaks simultaneously to an acknowledgment of vulnerability but also to the means of negotiating our responses (personal and collective) to the plurality of risks we are exposed to (Giddens, 2013). While trust in science and education does waver and differs across contexts and countries—it cannot meet every promise on every occasion or context—overall, it has remained relatively stable as evidenced by the endurance of its largely standardised and universalised content, curriculum and pedagogy.

Public trust in the formal education system has been earned by its success, by and large, in achieving its aims of educating the public. The education system does this through strong curriculum understanding that is built through careful consideration of what is important to know at any point in time and teaching expertise. Formal education has, therefore, largely fulfilled its social contract such that trusting in this system reduces risks. In turn, the formal education system depends on this trust—trust in teachers, principals, schools, universities, and educational governance. Trust allows educational organisations to further their own goals (Pepper et al., 2010). This trusting relationship has been shown to have a positive relationship on educational attainment (Browning, 2014; Dewulf et al., 2017). Education also plays a key role in transmitting and sharing knowledge, societal norms and expectations (Borgonovi & Burns, 2015) and is one of the strongest influences on social capital and increased trust (Borgonovi, 2012). The timescale over which this trust has been established in the formal education system is long.

Reliability—as a necessary component of trust—speaks to the stability of individual and collective action. This stability is necessary but not sufficient to establish trust. We can have predictable patterns of expectation that are met, but what is crucial to establishing trust is that those reliable systems and processes of action are rational: that is, grounded in judgements against an established set of criteria. Put another way, regularity and stability in forms of action count as trustworthy when they are judged to meet expected standards against a set of established criteria. Thus, we may speak of rationality as coupling reliability with actions in the world through agreements in the way we judge the status of something: say judging the truth status of a scientific claim in order to inform a decision. Stephen Mulhall (1994) puts this succinctly: “…what distinguishes rationality from irrationality in any domain is an agreement in – a commitment to – patterns or procedures of speaking and acting” (p. 26). Reliable commitments to norms, values and practices entail criteria for what is “appropriate”, but also the modes by which procedures of speaking and acting may be judged. It follows that trust depends also upon the reliability of the criteria against which we measure, or judge, whether a promise has been kept. That is, whether one’s words or actions are judged as having the status of a promise fulfilled—the status of trustworthiness. Different norms, values and practices will call for different criteria and hence establish different kinds of rationality around which trusting and trustworthy societies, communities and other collectives gather.

The advantage that this perspective on rationality, judgement and trust offers us is that we are not only able to recognise what makes science rational and hence trustworthy, but more importantly, acknowledge that science is only one form of rationality and hence one of a plurality of institutions and practices that engender trust. On the first point, Mulhall (1994) suggests that what is reliable and rational about science is a shared agreement amongst scientists about how to judge whether claims are well supported: that is whether the means by which a scientific claim or conclusion was reached were scientifically legitimate: Were the agreed upon methods of scientific justification and support followed, or were they not?

In the domain of logic and science, agreement between practitioners over the rectitude of a well-supported conclusion is agreed because disagreement with any legitimately supported conclusion is evidence of one’s incompetence in the practice; in other words such agreement merely signifies that the conclusion is legitimately grounded. (Mulhall, 1994, p. 27)

We acknowledge that such a view of science glosses over the on-going and well-established debates in the philosophical literature concerning the nature of the scientific method (see, for example, Sankey, 2013; Sober, 2015) as well as the extensive scholarship concerning how scientific methods are interpreted and applied in education (see, for example, Rudolph, 2005). Yet what is at stake here in this paper is consideration not of whether science is internally rational and consequently trustworthy, but rather how this rationality shows up in different contexts alongside other forms of rationality. Science, for its part, provides a mode of judgement that assesses the truth of beliefs, and it seeks to mitigate risk and uncertainty in circumstances where the status of truthfulness matters. When decision-making or problem-solving calls for judgements aimed at determining the truth, the methods of science have proven particularly well suited to reliably providing such judgements. However, reliability in establishing truth is only one way of grounding trust in action, speech and thought. Therefore, we are concerned more with the pragmatic consequences of the nature of science rather than philosophical discourse about the nature of science itself—as important as that is.

Trust in science cannot and should not be considered in absolute terms and independent of other forms of rationality. Not all actions or decisions in the world are, or ought to be, based on scientific knowledge alone—despite its proven reliability. For if this was the case, the absoluteness of scientific knowledge would threaten science’s commitment to openness and skepticism with respect to truth and expose the world to the combined risks of scientism and authoritarianism (Yaqoob, 2014). We must, therefore, account for other forms of rationality as well as the different contexts in which they may arise alongside or in competition with scientific rationality. The following section explores the forms of rationality that we use in this paper.

## Forms of Rationality

Trust in science emerges in any given context from its reliability in producing rational accounts of what is true. That is, science offers criteria against which to judge the truth-value of a set of claims. This in turn stems from a methodological commitment—an agreement—to practices and discourses of justification. In short, because science promises to deliver reliable and justified claims about the world and delivers on its promise, it is trusted. This of course relies on the assumption that what is called for on a given occasion and in each context is a commitment to truth and objectivity (Wilholt, 2013). More to the point, it is the nature of the occasion and the context that may contribute to whether there is such an expectation of science and that the objective truth science delivers can mitigate risk, in addition to establishing and maintaining social norms, social cohesion, and social capital. The corollary of this is that it is possible that contexts and occasions—such as the science classroom and the learning experiences therein—can be set up precisely to foreground the reliability and objectivity of science and promote a particular kind of trust in science. A science teacher, for example, may teach about climate change by addressing arguments made against the accepted scientific position on climate change. The teacher may ask the students to consider the claims being made (for and against the consensus view) and the evidence that supports these claims, and also the grounds for that evidence and assumptions that make the evidence count as evidence for the claims made (e.g. see Toulmin (2003) model of argument). This pedagogical approach foregrounds what makes science rational in the sense proposed earlier by Mulhall: that is to say, having a process for arriving at true, justified beliefs and having the means (the criteria) for judging whether claims are scientifically legitimate or not. Hence, education of this kind, which is supported by the formality and structure of the science classroom (e.g. lesson plans, curriculum expectations and assessment tasks), draws out the reliability of science and demonstrates that it delivers on its promise to distinguish truth from falsities.

The example above illustrates how judgments about what counts as scientific knowledge and what counts as legitimate practices or process that deliver scientific truth can be formally curated via classroom activities and discourses to privilege the reliability, rationality and trustworthiness of science. Yet other forms of rationality—reliable ways of establishing and agreeing upon ways of speaking and acting—are also in play in the science classroom, and in other contexts. Moreover, these forms of rationality are likely to interact—complementing or competing with one another in ways that affect the relative prominence of science’s trustworthiness. Consequently, we must take account of different kinds of rationality and consider whether the arrangement of these forms of rationality and judgements are characteristic of different spaces—spaces in which science and science education operate. To this end, and for the sake of simplicity, we introduce *propositional*, *procedural*, *perspectival* and *participatory* forms of knowledge and their associated forms of judgement (Gibson, 2014; Vervaeke & Ferraro, 2013) as a basis for understanding how rational encounters with and in the world are enhanced, and reliably so. Later we will examine how these provide a basis for rationality and agreements in judgement that may be helpful in identifying how trust in science is supported in three different contexts: formal, informal and casual.

Our earlier description of a classroom discussion dealing with climate scepticism already picks out the significance of propositional and procedural knowledge. Propositional knowledge, *knowing that*, is what commonly counts as knowledge in education. Although it may be taken as synonymous with facts, as previously discussed, it is properly knowledge that is judged to have the status of true, justified beliefs. In the case of climate science, this goes beyond learning or reciting facts about climate change, such as the increase in average global temperatures in the past two centuries. It includes knowledge of the (scientific) criteria and standards by which the truth status of facts and other claims to knowledge are judged. As part of a discussion of climate science, attention may also be given to whether students have adhered to the method of scientific reasoning and argumentation. That is, judging the application of a particular procedure or skill. Procedural knowledge is, therefore, knowledge of *how to* do things successfully and is judged by what advantage is leveraged by virtue of attaining high levels of competence in a skill. This may be a sophisticated skill like critical thinking or reasoning, or something like being able to do practical work in the classroom, write a report, or revise material for a science test.

Additionally, we have perspectival and participatory knowledge. Perspectival knowledge helps us recognise our social positions in a situation as well as our positions within shared practices. Hence, we need to be able to make judgements of what is relevant to particular situations, and to participate in the acquiring knowledge as a means of identity formation and being relevant in the world. For students in a classroom, perspectival knowledge amounts to knowledge of whether one feels “present” in classroom: that is, meaningfully engaged and aware of the educational situation. This is rational in the sense that being present or otherwise requires evaluation of one’s awareness in relation to the context in which one is embedded. Furthermore, because it relies upon self-awareness, this kind of knowledge helps shape one’s identity as a form of conscious perspective-taking. Participatory knowledge, on the other hand, is associated with the ability to recognise and attune oneself to the material and cultural environment. In the science classroom, this might include a student seeing an opportunity to learn a new skill or collaborate with her peers. It might also include a student recognising and taking an opportunity to impress her teacher by posing a sophisticated question. Hence, judging participatory knowledge—the kind and degree of participation—makes fitting in to the material and cultural environment a rational undertaking.

Judgements of propositional and procedural knowledge require, respectively, trust in one’s own knowledge and skills, as well as those of others. While the power of propositional knowledge is often foregrounded in education, the inclusion of procedural knowledge provides a way of demonstrating the reliability of the means of arriving at propositional knowledge. Perspectival knowledge engenders trust in one’s sense for “being there” and being present, and therefore is reliably and pragmatically relevant to our receptivity to the world. Trust engendered through participatory knowledge is related to this in that it captures the reliability of one’s capacity to see and take advantage of material and cultural opportunities (affordances in the language of Gibson, 2014) in the environment. What is key here is that trust, in the case of each knowledge form, grows the more one is required to make judgements or demonstrate that oneself or others are successfully employing the norms, practices and ways of being associated with each form of rationality.

There are likely other forms of rationality, other ways of reaching agreements in judgement that reduce the risk of misunderstanding about the material and social world, but also serve to re-affirm membership in a community and our presence in the world. Clearly, all forms of judgement are in play within the worlds of science and science education and hence trust in science and science education ought to be re-examined considering this. So, we turn now to using these forms of rationality to make a prima facie case for a distinction between formal, informal and casual spaces for science education and the consider the consequences for trust in science.

## Formal, Informal and Casual Science Education

Given the various places and avenues through which a young person learns about science or otherwise engages with science, it is useful to broadly distinguish between formal education and informal education. Formal education is acquired through the formal school system and curriculum. Informal education, as far as science education is concerned, comprises science education experiences outside formal schooling settings. These experiences involve a range of social groups, organisations and places that are institutional in character. The idea of informal science education recognises that “much of what people come to know about the world, including the world of science content and process, derives from real-world experiences within a diversity of appropriate physical and social contexts” (Dierking et al., 2003, p. 109). Informal science education has often been associated with science education that is organised by way of museums, science centres, extra-curricular science clubs and so on. Such informal spaces are seen to be “appropriate” complements to formal education owing to the presence of some trusted source to curate the science learning that is being offered in this space. Notwithstanding the role of family and the sociocultural setting in which young people grow up also has an influence on how young people identify with and know about science, we argue for an account of science education that considers a third space in which science learning takes place distinctive from both formal and informal contexts. It is a space that young people can access science information and understanding, but which is decentralised, casual and does not necessarily take the form of a physically limited space or a temporally limited event that someone might attend. This casual space was once dominated by mass, broadcast media and publishers, but is now predominantly a space of online communication consisting of a broad range of information sources. Like the informal space, some of these sources are deemed “appropriate” in that there is a trusted organisation or knowledgeable person developing and disseminating materials. However, the deregulated and openness of this space means that many sources do not come with such assurances. In what follows, we will explore the characteristics of each space in relation to trust and outline their respective and interrelated contributions to science education.

The purpose of formal science education or school science, while debated, can be described as teaching students about conceptual and theoretical knowledge, the nature and methods of science and its development, as well as the process of doing science (Hodson, 1998). It has been noted that these purposes have been built into the science curriculum over many decades (Hodson, 1998; Stocklmayer et al., 2010). While there is also a view that science needs to be able to respond to a changing social world and that science curricula need to be constructed in a way that enables “young people to engage with science-related issues that are likely to be of interest and concern to them” (Jenkins, 1999, p. 707), formal curriculum development by its very nature is slow to respond to such calls. So, while the claim that the science curriculum in most countries is highly centralised has been well considered, vetted and debated—and represents our best understanding of the science covered at the time of implementation—the lead-in time for curriculum change required in most education systems is on the scale of years to decades. And while many teachers are adept at retrofitting the curriculum to meet the needs of their students, this is not the path that all teachers take. Similarly, science education in the formal and informal space has accrued both cultural value and significance that calls simultaneously for conservation about what has been established in curriculum and a process of renewal in line with shifts in social and cultural norms and values.

Science education also sits outside of school education in a broad range of informal settings. There are museums, festivals, community organisations and informal and extra-curricular school activities. There has been discussion in the literature that attempts to differentiate between formal and informal education (Stocklmayer et al., 2010; Wellington, 1990). These often focus on differences in the learning that takes place in formal and informal settings. These discussions emphasise student choice, open curriculum, context and relevance (Malcolm et al., 2003). These informal spaces also present a connection to the cultural material world (Mulcahy, 2018). In these informal settings, there is not necessarily a link to the formal curriculum or schooling, and it is less centralised. This means that the science that is communicated and taught in these settings can be more responsive to more urgent needs and gaps in the market as they arise. However, the learning experiences are for the most part, designed and curated by organisations that have some specialist science knowledge. Decisions about what is “appropriate” to include in these informal settings is set by an organisation and the demands and constraints of its institutional agenda and aims.

The final setting for science education we refer to is the casual space. This space is often included, without careful distinction, within discussions of informal education. However, we propose that this space warrants analysis and study as a distinct category. For instance, while this space has some overlap with informal settings with respect to a learner’s choice, the choices available in the casual space are categorically different because they are in some sense open and unlimited. While content in the informal space is shaped around what is “appropriate”, curated and institutionalised, this is not necessarily the case in the casual space. The casual space, while also incorporating sources that are seen as “appropriate” by formal and informal institutions, is highly decentralised and open to all contributions. This means that in the casual space, there is not a restriction on norms as there are many on offer. One can just as easily find themselves looking through material in the casual space that is scientifically accurate as material which is, deliberately or unintentionally, misleading. It can nevertheless be construed as educative in the sense that those accessing this information believe that they are learning something new, regardless of whether it is accurate or not. We also know that this space is increasingly used by young people to access information (Wellcome Trust, 2013) especially where a particular need for information is not being met elsewhere: for instance, from family and friends or informal and formal education settings. Moreover, this casual setting can be immediately responsive to current situations and is very much a space where young people are intrinsically motivated and have agency over what information they access.

In this paper, we posit that the science on offer in these three different educative spaces is accessed differently by young people and that this third, casual space needs to be paid particular attention given the nature of the knowledge in that space and how people make judgements about the science they are accessing. It is here that issues of trust arise. The distinction between these spaces provides a useful analytical framework for considering the different forms of rationality at play when information about science arises in different settings. What constitutes trust in science in these spaces and how does it function? How can we enable young people to discern and judge what is and is not trustworthy in the casual space? We respond to these questions in the following sections as we explore the forms of rationality that are foregrounded in each of the three spaces of science education. In doing so, we acknowledge that we are treating these three spaces as ideal types whereas in reality there is overlap between the spaces. Our aim in separating the spaces is to consider which forms of rationality are most emphasised rather than suggesting other forms are not present.

## Trust in Science in the Formal Education Space

This section explores the role of trust in formal science education. As discussed in the previous section, formal science education is by and large constructed around well established and reliable science. In order to understand the forms of rationality that are prioritised in formal science education, we will consider the knowledge that is included in the school science curriculum. Curriculum reflects “what counts” as valid knowledge at a particular time (Bernstein, 1971). While there are national differences in science curricula, by and large science curricula have significant overlap (Schmidt et al., 2005). This reflects the propensity of science to move, over time, toward singular truths and theories that account for the natural world. As such, curriculum documents tend to list well recognised reliable science content. Established science content is just one component of the propositional knowledge that is included in the science curriculum. Increasingly a consideration of the nature of science, including how claims should be judged, has also been included as a component of propositional knowledge in the science curriculum (Rudolph, 2005).

Alongside propositional knowledge, school science has a significant focus on procedural elements of science. The practice of undertaking practical work and writing science reports are common examples used in science classrooms as a demonstration of how science is undertaken (Abrahams & Millar, 2008). This requires the ability to undertake procedures in alignment with the norms and practices of the scientific method. Formal education therefore places emphasis on judging whether propositional knowledge is sound and the ability to make judgment of whether the correct procedure was followed.

It has been argued that school science tends to present a “rather simplistic, narrow and unproblematic account of science” that “rarely coordinates the epistemic, cognitive and social dimensions of science” (Erduran and Dagher, 2014, p. 2). While the place of propositional and procedural knowledge is well established in science education, what is less explored are participatory and perspectival knowledge. Those forms of rationality provide an awareness of one’s context and degree of ‘fit’ within one’s social-material environment. While participatory and perspectival knowledge are present in science classrooms, and more obvious in some classrooms than others, these forms of rationality are less obvious.

Thus, trust in science promulgated in the formal space becomes speciously connected to the reliable coupling of doing and knowing more so than the connection between science and an individual’s identity and awareness of fitting into the world. This suggests that trust in science is limited in formal science education (so conceived) by two constraints: firstly, the controls imposed upon science education by the methods of science employed; and secondly, the restriction to propositional knowledge. In practice, these constraints are enacted through curriculum choices and pedagogical approaches.

## Trust in Science in the Informal Education Space

The space in which science education takes place informally, in museums, science centres, zoos, science galleries, after school programs etc., picks out and amplifies a different sub-set of rational encounters that promise other justifications for trust as well as community building. While there is a commitment to grounding a person’s encounter and engagement with science as a set of pre-judged, true, justified beliefs, informal education settings aim for (i) experiential and embodied emersion in science in combination with (ii) an engagement with science as a material-cultural environment (Mulcahy, 2018). The first of these calls to mind perspectival knowledge, and the trust in science that is afforded by the sense that participants are present to science-as-doing, -seeing, -touching, -hearing, -feeling, etc. Granted, this is available in formal science education too, yet for the informal setting, it amounts to a *raison d’etre*, and in some ways foregrounds the idea of fostering and building *public* trust in science. The second, which attends to participatory knowledge, deals instead by facilitating and fostering the fit between the public and the culture of science as well as the cultural significance of science. Informal science education settings foster and promote the reliability with which science affords social, cultural, economic, technological and political possibilities for action. Consequently, informal science education must couple its curation of propositional knowledge, with a heightened responsiveness to the present cultural status and environment of science, which may include contemporary and cutting-edge research and debates, crises and controversies. In this respect, it also draws much closer to addressing an individual’s sense of curiosity and perplexity: that is, it aims to foreground a worldly experience of science rather than merely “knowing” and “doing” science for their own sake.

What we have posited thus far is the degree to which formal and informal domains of science education not only foreground particular forms of rationality and judgment, but also cement these within institutional practices and norms that offer the stability and assurance necessary for the development of trust in science. In the case of formal education, this shows up as the formal curation of scientific knowledge and thus privileges propositional knowledge. Within informal settings, the vehicle for science education is primarily experiential and cultural and so in the informal space perspectival and participatory knowledge are brought to the fore.

## Trust in Science in the Casual Education Space

The third science education space, the casual, is characterised by the absence of overarching, institutionalised practices and norms that characterise the formal and the informal education spaces, as well as the largely guaranteed reliability of the knowledge that is presented. The casual space is the home of multiple norms and truths. Rather than a unified, stable, independent, uniform, universal and culturally accepted set of agreements in judgement or preferred and settled forms of rationality, the casual space is one in which individuals have, in principle, potential access to all forms of rationality and judgement—including those we associate with the formal and informal domains.

In practice, the casual space consists of young people’s access to a myriad of local “worlds” vying to secure stability and to gather a trusting and trustworthy community. These worlds may be accessed by people searching for a particular kind of knowledge that a given world or community offers; or they may be experienced through direct membership of a community that shares converging interests, values, practices, knowledge and so on. Examples include online communities on social media platforms, content creation platforms and online gaming platforms. These worlds also include curated knowledge spaces like online encyclopaedias and search engines in which editorial communities or search algorithm designers set up the parameter space of what knowledge is available to individuals. Additionally, young people have access to online educational spaces that cater simply to skill development, right up to complex university-level coursework. Added to this is an unprecedented access to digitised texts and cultural artefacts, including artworks, performances and music from different times, places and cultures. Moreover, this space gives young people more rapid—although not necessarily unbiased—access to news and current events, including developments in science. The casual space also offers a communication platform for providers of informal science education, like museums, science centres, publishers and public or private scientific institutions. Finally, this space is one that includes information about science that is problematic, including sites that deliberately set out to be anti-science but also those that unknowingly are communicating versions of science that are incorrect.

Each of these worlds within the casual space—these gatherings of people and forms of knowledge—is a response to disparate and sometimes conflicting or contradictory interests. A discussion board may gather members interested in advancing specialist scientific or technical knowledge, whereas other communities may gather and draw young people interested in the more aesthetically engaging aspects of science, such as demonstrations, gadgets and experiments, or more accessible visual and narrative explanations of scientific concepts. Equally some may capitulate versions of science that are problematic. In these worlds, and in relation to these worlds, the ideas of following and influencing, or of content creation and content consumption, alter the meaning of learning or teaching. Yes, propositional scientific knowledge is accessible through, and can be built within communities in the casual space. So too can general or specialised skills be demonstrated and developed in groups in the casual space and accessed by young people on a casual basis: bypassing the temporal and physical constraints of formal and informal education. However, the reliability of the propositional and procedural knowledge in the casual space is not determined by some overarching structure or standard of judgement, which is more readily available—for better or worse—from institutionalised power, norms and practices.

Trust in science as it emerges in the casual space must be established anew within each world, according to the interests and risks that its members face together. Furthermore, there is no guarantee that those interests and the worlds they gather will endure. The casual space is much more dynamic than the formal and informal. Trust is tentative: stable enough. Hence, perspectival and participatory forms of knowledge and rationality play a very important role in the casual space. Participants in such communities, as well as those accessing the knowledge available in the casual space, must be sensitive to the situation that gives rise to, and helps sustain these communities. This may be a scientific controversy for instance, or an alternative take or interpretation of the science, or its outright rejection. That is, the casual space provides access to more knowledge and skills, but what makes these trustworthy is the degree to which they are perceived as affording actions that reliably serve the needs of individuals. Moreover, the reliability of what is offered within the casual space, what is worthy of trusting, depends also upon the degree to which an individual sees themselves fitting in a world with others—and this includes worlds in which science may or may not be trusted. In this respect, the casual space provides many more opportunities or options to find worlds that fit the individual—contrasting with the task of fitting oneself to “the” world. This makes trust more available in the casual space in so far young people can entertain more perspectives, and because the proliferation of “worlds” increases the likelihood of finding a culture in which participation is comfortable, reliable and trusting. Yet it also makes it more elusive given that the problems and projects around which these worlds gather are constantly evolving. Indeed, the trusted world of science is just one of many. Hence, the casual space has the advantage over the formal and informal spaces of better distributing risk—even as this ushers in its own kind of risk.

## Discussion

We have argued that young people’s engagement with science and science education, broadly speaking, operates across three spaces: formal, informal and casual. These findings are summarised in Table 1. The formal domain is one in which the reliability and trustworthiness of scientific (propositional) knowledge, along with its modes of production and construction (procedural), is foregrounded. The informal is distinctive in prioritising an experiential (perspectival) and material/cultural (participatory) emersion in a reliable construction of science. Both formal and informal domains feed into, and feed off, the reliability of science and therefore take up the mantel of trusted institutions. The casual space, while being able to accommodate these institutional practices (and the trust, reliability and social capital they afford), does so in relation to other forms of rationality and judgment. So, for instance, scientific knowledge communicated by experts from informal science education institutions may have a smaller share of an online audience than an influential content provider despite the weight of trust placed in the institution. Or the accuracy of a science teacher’s pronouncement may be checked against online sources. As such, the aspects of judgement in the casual space might be taken as competing with the formal and informal spaces for the cultivation of trust. Alternatively, it may assume a complementary role that underscores and acknowledges the complexity of the context, and communities, in which trust in science commonly arises. There are likely to be times where the stability of scientific knowledge is challenged, where the science is still emergent such as over the COVID-19 pandemic. Here the rate at which scientific ideas are changing or perhaps more importantly, perceived to be in a state of flux, provides an additional challenge to the perceived steadiness of forms of rationality and judgement privileged in institutional spaces.

**Table 1:** Framework summary for formal, informal and casual science education

Dimension	Formal space	Informal space	Casual space
De/centralised	Centralised	Centralised	Decentralised
Regulation	Regulated	Regulated	De-regulated
Timescales	Long timescale Incremental change	Long and medium timescales	Short, dynamic timescales
Dominant and (Subdominant) modes of rationality and judgement	Propositional Procedural (Perspectival) (Participatory)	(Propositional) (Procedural) Perspectival Participatory	Propositional Procedural Perspectival Participatory

In science emergencies such as COVID-19 and climate change, it may be that the science appears unsettled or falls short of meeting the demands of individuals or communities. As recent times have shown, such emergencies or crises come into their own in the casual space and as Arendt puts it “A crisis becomes a disaster only when we respond to it with preformed judgements, that is, with prejudices” (Arendt, 1958, p. 493). The casual space is where people turn for information in everyday life and particularly when there is an absence of other reliable information. The distinction between the three spaces that has been set out provides opportunities to reconsider what science education in the formal and informal domains has to offer the casual space when people access this space for science knowledge in everyday circumstances and in science emergencies. Equally however, there is much that the casual can contribute to formal science education and institutional practices and contexts.

We have posited that the three spaces under discussion pick up and amplify certain forms of rationality over others, and thereby gather communities that find security and build trust in science using what each mode of rationality and domain offers. We see that these spaces may also preferentially select for the various dynamic timescales over which scientific claims to knowledge appear stable, and hence trustworthy. The formal space couples its foregrounding of propositional knowledge with the reliable march of scientific progress. The informal space concerns itself with the experience of science as embedded in the fabric of contemporary cultural life, so it is much more sensitive to the recent past and notable breakthroughs and discoveries. The casual space also makes the slow and steady advance of science and the regular scientific breakthroughs available to the individual and the public at large. However, the decentralised, de-institutionalised, distributed, highly interconnected and speedy potentiality of the casual space also provides access, particularly in times of emergency, to the rapidity of emerging scientific knowledge. This contributes to a sense of uncertainty about scientific knowledge that may appear unsettling and risky, and therefore is likely to promote the search for reliability and trust in other registers. The largely open and free information channels, along with the rise of the self-made specialist and influencers, provide an environment with the potential—certainly on short timescales—to help stabilise the truth or destabilise the truth, in equal measure.

Both formal and informal educational spaces benefit from the authority that comes from the slow and reliable construction of canonical scientific knowledge. The stability and reliability of science comes not from the durability of the conclusions per se: scientific knowledge is not static. Rather, according to Mulhall (Mulhall, 1994, p 27.), what gives science its reliability is a shared agreement on the range of permissible methods. That a layperson or expert challenges the conclusions reached by a climate scientist, for example, is inconsequential as far as science is concerned, unless what is in question is whether the scientist was consistent and reliable in the execution of the methods that led to those conclusions. In this view, “trusting the science” ought to coincide with trusting that the scientific methods were applied faithfully. Securing trust in science in the formal and informal spaces is an easier task because constraining the curation of scientific knowledge and the cultural significance of science makes it easier for people to infer a singular and stable origin to scientific knowledge and its cultural significance. Because the casual space has increased temporal responsiveness to risk, it is more likely to privilege product over process. That is, the casual space is more likely to favour appropriation of scientific conclusions (facts, theories and findings) that most immediately serve the interests and choices of individuals and communities, as they rapidly evolve. What is missing from the casual informal space are the criteria by which to judge conclusions scientifically justified with respect to method, rather than simply in relation to other, often competing or contradictory “scientific” conclusions. The formal and informal educational contexts, therefore, have an important role to play in exploring and modelling this unique—methodologically dogmatic—aspect of science.

The dynamism, freedom and breadth of choice in the casual space (from amongst the myriad complementary ways in which science can show up) provide a counterpoint to the taken-for-granted trust in science that is largely secured in the formal and informal settings. The kind of science education available in the formal and informal spaces is contingent upon the stability of science in relation to agreements in judgements of claims to truth—that is, it secures epistemic trust. Science education perhaps enjoys a double blessing of the public’s trust in education and trust in science: the former arising from education’s delivery on much of its promises (e.g., Labaree’s (1997) social efficiency, social mobility and democratic equality), and the latter relying upon the reliability, or promise of reliability, invested by or inherited from the objectivity (real or perceived) of the sciences.

Yet securing claims to objective knowledge is not the only means of establishing trust in science. It is instructive to examine how judgments can function to gather a trusting community around trustworthy people or institutions while forgoing the constraints around methodology and objectivity imposed by science’s commitment to truth seeking. We see this most clearly in aesthetic judgments. Mulhall describes it thus:

Aesthetic debate is thus a way of constructing or discovering community through the articulation and development of individuality; it shows a way in which community can be founded upon the fuller expression, rather than the complete repression, of individuality. The possibility that we may find limits to that community is the price to be paid for the possibility of creating that community without sacrificing subjectivity. The fact that such a community of response and thought is not guaranteed shows something about the sort of community it is – one in which membership is freely willed, elicited rather than compelled from each individual. If this sort of community can result only from abandoning the guarantee of agreement, then it is hardly surprising that we sometimes choose abandonment; and with such a vision to prompt us, the risks involved in attempting to achieve it (humiliation, rebuff, the discovery of isolation) may seem well worth running. (Mulhall, 1994, pp. 28-29)

While scientific communities are gathered by and discriminate based on a methodological commitment to objectivity, many people outside of science make aesthetic judgements that nevertheless establish trust by finding that their articulation of their subjective life comes to speak for the subjective experiences of others. The subjectivity here is not irrational. It is subjective yet forms the basis of trust since it establishes reliable aesthetic categories that may be shared with others. Moreover, because the demands of aesthetics are less constrained than those of science—more forgiving and responsive to change—it resists the categorical and potential totalitarian tendencies of science. Such aesthetic subjectivity is certainly in play in the three spaces, but in the formal and informal spaces, aesthetic judgements are also likely to be circumscribed by the aesthetics of science: the view that scientific objectivity is the final arbiter of truth can, after all, function as a subjective and aesthetic claim.

We must, therefore, be mindful, that in all three spaces – but particularly in the casual – the propositional knowledge constructed by science (via its objectivity, and its methodological commitments to truth seeking that demarcate scientists from non-scientists) is also available by exercising our subjectivity (through aesthetic judgments; including aesthetic judgments of science itself). At its worst, an aesthetic appropriation of science is a form of scientism: parasitising the trust in science in ways that ignore or eschew the objective constraints of science. At its best, it is an acknowledgment of the need for individuals and communities to make meaning with whatever (rational) resources are available. Education in the casual space makes much more available in addition to the promises of science and science education.

## Conclusion

This paper has noted the importance in science education of proposing a third space—the casual space—alongside formal and informal education, that is underdeveloped in extant science education research. This space sits outside of the formal, institutionalised and carefully curated spaces of formal schooling and informal education. This casual space is increasingly important to society and the space where people most often turn to seek out information about science. The casual space is agentive and driven by self-motivation. However, the development of trust in science in this space differs from that in formal and informal science education due to the vast range of information, truths and norms available.

This paper explored trust in science with respect to the different modes of knowledge and rationality that are at play in the formal, informal and casual spaces wherein science is accessed. Through a consideration of the modes of rationality that are emphasised in these three different science education spaces, we have investigated the role of judgement as a means of assessing truth, skills, perspectives and participation. In doing so, we show that while these three spaces all include propositional, procedural, participatory and perspectival knowledge, each space emphasises certain forms of rationality over others. Around these forms of rationality and the judgements that are made in each space, communities are formed that find solace in the norms, values and practices these rationalities offer. The casual space is characterised by the formation of different communities, gathered by a need to acknowledge the risks that attend them, and the knowledge that mitigates such risks. So, while there is trust in science, and the link between scientific claims and scientific method particularly in the formal education space, we recognise that the judgements being made in the casual space may be more aesthetic and subjective, and therefore more open to the risks that objectivity seeks to mitigate.

Formal education has an important role in establishing and maintaining trust in science. What gives the formal space its formality is the controls and constraints on content and modes of rationality, so it becomes a safe and stable space in which to incubate trust. If a learner can establish trust in science in this contained space, supported by educators, it may provide a model or exemplar to take into other domains and tested against other forms of rationality. It therefore needs to be a space in which we help students develop an understanding of how to make judgements while acknowledging the role of agency, context, preformed judgments and aesthetics.

The growing significance of the casual space, as posited here, is one that cannot be ignored. It has many characteristics and bases for engendering trust in science that could benefit both formal and informal science education. This includes the ways in which people engage with and seek information in the casual space, and especially the way the casual space can respond with immediacy to emerging developments in science. The restraints of formal education also limit engagement with many science topics. While informal education is less tied to a curriculum, it also does not seem to engage as readily with emerging and topical science in the same way the casual space does. Perhaps, what is required is a more responsive formal and informal science education that fills the gap that is currently only being addressed in the casual space.
